# Predictive Markers of First Line Pazopanib Treatment in Kidney Cancer

**DOI:** 10.1007/s12253-020-00853-9

**Published:** 2020-06-22

**Authors:** Zsófia Küronya, Mihály Dániel Szőnyi, Krisztián Nagyiványi, Fruzsina Gyergyay, Lajos Géczi, Barna Budai, Tamás Martin, Andrea Ladányi, Edina Kiss, Krisztina Biró

**Affiliations:** 1 Department of Genitourinary Medical Oncology and Clinical Pharmacology, National Institute of Oncology, Ráth György utca 7-9, Budapest, 1122 Hungary; 2 Semmelweis University, Budapest, Hungary; 3 Department of Molecular Genetics, National Institute of Oncology, Budapest, Hungary; 4 Department of Surgical and Molecular Pathology, National Institute of Oncology, Budapest, Hungary; 5 Medical Centre, Hungarian Defence Forces, Department of Oncology, Budapest, Hungary

**Keywords:** Kidney cancer, Pazopanib, Liver metastasis, Side effects, Efficacy

## Abstract

Real-world evidence from clinical practices is fundamental for understanding the efficacy and tolerability of medicinal products. Patients with renal cell cancer were studied to gain data not represented by analyses conducted on highly selected patients participating in clinical trials. Our goal was to retrospectively collect data from patients with advanced renal tumours treated with pazopanib (PZ) to investigate the efficacy, frequency of side effects, and searching for predictive markers. Eighty-one patients who had received PZ therapy as first-line treatment were retrospectively evaluated. Overall survival (OS), progression-free survival (PFS) were assessed as endpoints. Median PFS and OS were 11.8 months (95% CI: 8.8–22.4); and 30.2 months (95% CI: 20.3–41.7) respectively. Severe side effects were only encountered in 11 (14%) patients. The presence of liver metastasis shortened the median PFS (5.5 vs. 14.8 months, *p* = 0.003). Median PFS for patients with or without side effects was 25.6 vs. 7.3 months, respectively (*p* = 0.0001). Patients younger than 65 years had a median OS of 41.7 months vs. 25.2 months for those over 65 years of age (*p* = 0.008). According to our results absence of liver metastases, younger age (<65 years) and presence of side effects proved to be independent predictive markers of better PFS and OS.

## Introduction

Renal cell cancer represents only 2% of all cancer diseases [1], however, it comprises 90% of all renal-originated tumors [2]. 25–30% of patients already have distant metastases at the time of diagnosis, and further 25–30% of those who initially are resectable eventually recur and develop distant metastases [2]. Consequently, the overall five-year survival is as low as 20–25%. [3].

The breakthrough in the treatment of renal cell carcinoma (RCC) was the introduction of small-molecular-weight tyrosine-kinase inhibitors (TKI) of the vascular endothelial growth factor (VEGF) signalling pathway (sunitinib, sorafenib, pazopanib), which inhibit the intracellular domain of VEGF receptors. Although the range of therapeutic options has broadened due to the introduction of immunotherapeutic modalities and their combinations, these medications will continue to play a crucial role in the treatment of renal tumours and their future status will be determined by ongoing clinical trials.

Pazopanib is an oral, multi-targeted tyrosine kinase inhibitor with effect on VEGFR, platelet-derived growth factor receptor (PDGFR) and the c-Kit tyrosine kinases [4]. In a phase III clinical study, 450 patients with or without previous cytokine therapy were randomized to pazopanib and placebo treatment arms [5]. Patients had mostly good or intermediate prognosis. Pazopanib significantly increased the progression-free survival (PFS) compared to placebo (9.2 vs. 4.2 months, HR: 0.46, 95% CI: 0.34–0.62). This increased PFS was noted in both groups of patients, with or without previous treatment. An improvement in OS was also achieved (21.1 vs. 18.7 months), but the difference was not significant as cross-over was allowed from placebo to pazopanib arm (HR in mortality was 0.91, 95% CI 0.71–1.16) [6]. The treatment was well tolerated, the most common side effects, similarly to the other TKIs, were diarrhoea, hypertension, hair colour change, nausea, anorexia and vomiting. Clinically significant elevated liver function developed in 10% of patients with occasional dose reductions being necessary, however, haematological toxicity was observed only in 1% of patients. Based on the results of the phase III study, pazopanib has been approved by the US Food and Drug Administration (FDA) in October 2009 [7], and in February 2010 it gained European Medicines Agency (EMA) approval as well [8]. In a subsequent, phase III non-inferiority COMPARZ study, pazopanib was shown to be comparable in efficacy to sunitinib [9, 10], and in a quality of life (HRQoL) comparative study (PISCES), pazopanib was proved to be better tolerated than sunitinib [11].

A large patient database was studied retrospectively, with the current study aiming to define patient traits contributing to effective pazopanib treatment, based on everyday clinical practice. The purpose of the study was to analyse efficacy and tolerability of pazopanib, with the aspiration of determining the factors responsible for longer PFS and OS.

## Methods

### Patients

In the present study, we processed data from 2013 to 2019 on renal tumour patients treated with PZ at the Department of Genitourinary Medical Oncology and Clinical Pharmacology of the National Institute of Oncology. The study was approved by the Medical Research Council: (21679–2/2016), and by the Ethical Committee of the institute.

Between 11th May 2013 and 13rd March 2019, 157 patients had their PZ treatment initiated. Out of the 157 patients, we evaluated the data of the 81 patients who received PZ treatment in the first line setting, who underwent nephrectomy and had pure clear cell histology.

For the retrospective data analysis patients’ data were extracted from the electronic database of the institute. In all cases, the initial dose of PZ was consecutively 800 mg/day. Dose reduction or delay was performed at the oncologist’s discretion, based on the severity of side effects. Treatment was continued until disease progression based on RECIST 1.1 (Response Evaluation Criteria in Solid Tumors), intolerable toxicity, or until death. Tumour response was assessed by CT or MRI every 12 weeks, and adverse events were evaluated according to CTCAE 4.0 and 5.0 (Common Terminology Criteria for Adverse Events).

### Statistical Analysis

PFS was calculated from the start of the PZ treatment until disease progression or death, or, if there was no progression, until the last follow-up date, while OS was calculated from the start of the treatment until death or the end of the follow-up period. PFS and OS were evaluated by Kaplan-Meier method and log-rank test. Parameters significant in univariate evaluation were selected for multivariate Cox regression analysis. For the significance level *p* < 0.05 was applied. All analyses have been conducted with the NCSS12 software [NCSS 12 Statistical Software (2018), NCSS, LLC. Kaysville, UT, USA, ncss.com/software/ncss].

## Results

### Patient Characteristics

Patients’ characteristics were detailed in Table 1. The mean age of patients was 65.3 years (range 40–85 years) and the male to female ratio was 1.9:1. Prognosis has been graded according to the Memorial Sloan Kettering Cancer Center (MSKCC) guidelines [12] (Table 1). Most of the patients belonged to the moderate prognostic group, while the second largest group had good prognosis. All patients underwent nephrectomy and 19 patients (23%) had metastasectomy (Table 2). In 36 cases, the metastases were identified along with the diagnosis of the primary tumour (synchronous), meanwhile in 45 cases, it developed later (metachronous) (Table 1).

**Table 1:** Detailed patients’ characteristics

Patient characteristics	*N* (%)
Age (years)	
Average	65.3
Median	67
Range	40–85
Gender	
Male	53 (65)
Female	28 (35)
Histology	
CCRCC	81 (100)
Prognosis	
Favourable	35 (43)
Moderate	37 (46)
Poor	7 (9)
N/A	2 (2)
Nephrectomy	
Yes	81 (100)
Metastasis type	
Synchronous	36 (44)
Metachronous	45 (56)
Location of metastasis	
Bone	31 (38)
Lung	49 (60)
Brain	9 (11)
Lymph node	23 (28)
Pancreas	6 (7)
Liver	7 (9)
Pleura	6 (7)
Skin	4 (5)
Thyroid	3 (4)
Adrenal	11 (14)
Other	2 (2)
Local recurrence	9 (11)

*CCRCC* clear cell renal cell carcinoma, *N/A* not available

**Table 2:** Details of pazopanib therapy, additional and subsequent treatments

Length of pazopanib therapy	Months
Average	16.1
Median	8.7
Range	0.3–103.9
Dose reduction	*N* (%)
No	50 (62)
Yes	20 (25)
Discontinuation	11 (14)
N/A	3 (4)
Pazopanib therapy still ongoing	*N* (%)
Yes	19 (23)
No	62 (77)
Tumour response	*N* (%)
Complete response	9 (11)
Partial response	24 (30)
Stable disease	28 (35)
Progressive disease	6 (7)
N/A	14 (17)
Subsequent treatment lines	*N* (%)
None	53 (65)
Everolimus	10 (12)
Axitinib	1 (1)
Nivolumab	10 (12)
Sunitinib	5 (6)
More than two lines	4 (5)
Additional treatments	
None	26 (32)
Radiotherapy	33 (41)
Metastasectomy	19 (23)
Radiosurgery	5 (6)
Adjuvant interferon	5 (6)
Bone surgery	3 (4)
Spine surgery	3 (4)
Embolization	1 (1)
N/A	8 (13)

*N/A* not available

### The Efficacy of the Treatment, Predictive Factors, and Side Effects

The mean duration of pazopanib therapy was 16.1 months (median 8.7, range 0.3–103.9 months). Dose reduction due to adverse events occurred in 20 cases (25%), treatment discontinuation took place in 11 cases (14%) (Table 2). At the end of follow-up on October 1, 2019, seventeen patients were still receiving PZ therapy, the remaining of the patients had the treatment stopped mainly due to progression (*N* = 48) or death (*N* = 16). The best response was complete remission (CR) in 9 (11%) cases, partial remission (PR) in 24 (30%), stable disease (SD) in 28 (35%), and progression developed in 6 (7%) cases. Fourteen patients (17%) were not evaluated, or the assessment was not available. Further treatments and additional therapies following PZ therapy are also listed in Table 2. Severe, grade 3/4 side effects were rarely presented, only in 11 (14%) patients, meanwhile, 32 (39%) of patients did not present any adverse event. Adverse events are detailed in Table 3.

**Table 3:** Side effects of PZ treatment recorded during the study period

Side effects	Grade 1–2, *N* (%)	Grade 3–4, *N* (%)
Patients with side effects	38 (47)	11 (14)
Diarrhoea	28 (35)	2 (2)
Fatigue	14 (17)	0
Hypertension	11 (14)	1 (1)
Mucositis	9 (11)	0
Nausea, vomiting	8 (10)	1 (1)
Skin problems	8 (10)	0
Decreased liver function	6 (7)	3 (4)
Cardiovascular toxicity	3 (4)	5 (6)
Decreased kidney function	3 (4)	0
Hypothyreosis	2 (2)	0
Haematological toxicity	1 (1)	1 (1)

**Fig. 1:**
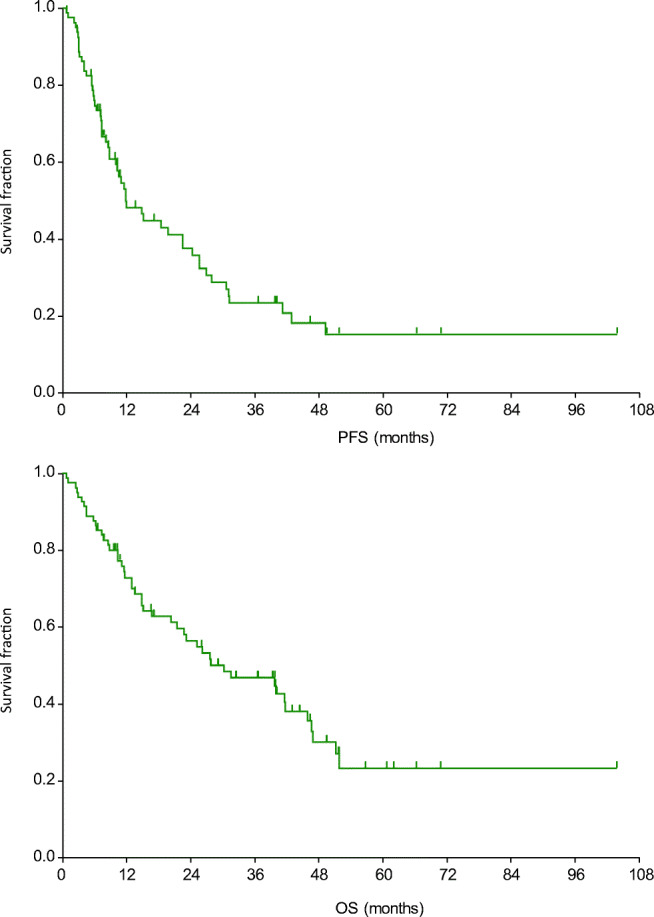
Progression-free survival (PFS) and overall survival (OS) curves of all patients. Median PFS: 11.8 months (95% CI: 8.8–22.4); median OS: 30.2 months (95% CI: 20.3–41.7)

**Fig. 2:**
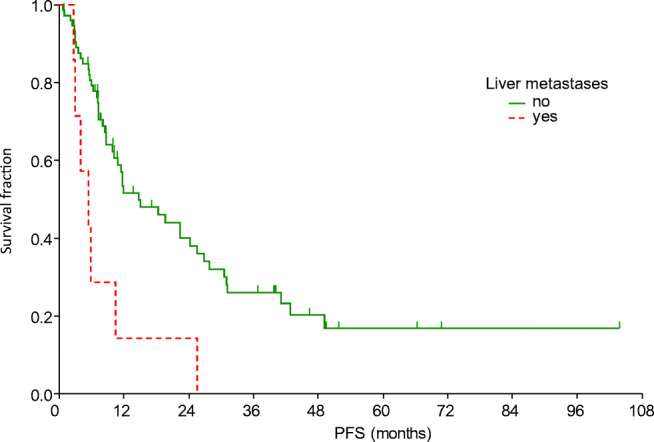
Progression-free survival curves according to the presence of liver metastasis

**Fig. 3:**
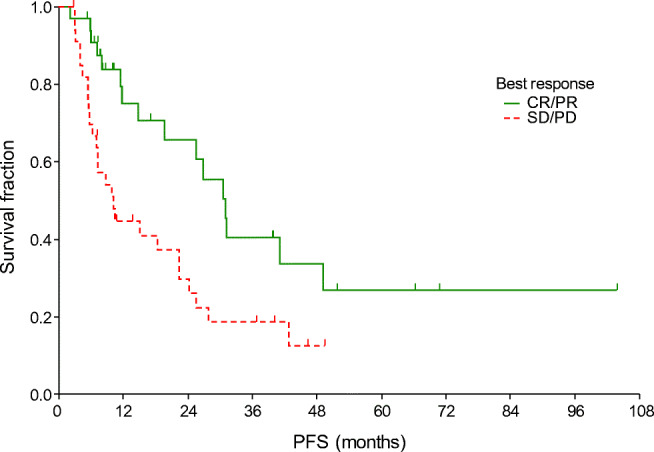
Progression-free survival curves according to treatment response. CR, complete remission; PD, progressive disease; PR, partial remission; SD, stable disease

**Fig. 4:**
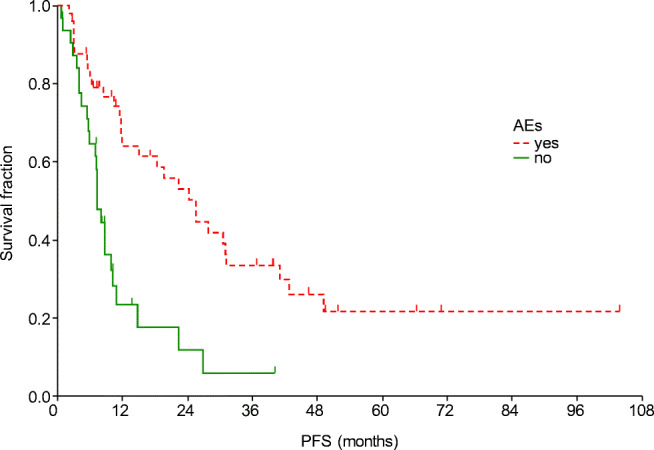
Progression-free survival curves according to the presence of adverse events (AEs)

After a median of 43.0 months of follow up, the median PFS was 11.8 months and the median OS was 30.2 months (Fig. 1). The median PFS was shorter in patients with liver metastasis (Table 4, Fig. 2). Moreover, PFS was found to be shorter in patients without remission or side effects (Table 4, Figs. 3 and 4). Dose reduction and among side effects, diarrhoea proved to be predictive factors, however they were not independent markers of PFS (Table 4). In univariate analysis, PFS was found to be longer in patients who underwent dose reduction (Table 4).

**Table 4:** Uni- and multivariate analysis of progression-free and overall survival

Parameter	mPFS (95% CI)	p_LR_	OR_Cox_ (95% CI)	p_Cox_
Liver metastasis				
Yes	5.5 (3.0–5.9)	0.003	1 (reference)	0.0004
No	14.8 (10.2–24.4)		0.18 (0.07–0.47)	
Tumour response				
SD/PD	10.2 (7–22.4)	0.007	1 (reference)	0.024
CR/PR	31.1 (19.7–41.2)		0.48 (0.25–0.91)	
Side effects				
Yes	25.6 (12–31.1)	0.0001	1 (reference)	0.003
No	7.3 (5.9–8.8)		2.88 (1.44–5.74)	
Parameter	mOS (95% CI)	p_LR_	OR_Cox_ (95% CI)	p_Cox_
Age				
<65 years	41.7 (23.2–51.9)	0.008	1 (reference)	0.007
≥65 years	25.2 (11.1–30.2)		2.7 (1.31–5.53)	
Liver metastasis				
Yes	11.1 (5.8–14.8)	0.007	1 (reference)	0.004
No	39.8 (23.2–46)		0.21 (0.07–0.6)	
Tumour response				
SD/PD	25.2 (14.8–30.2)	0.008	1 (reference)	0.01
CR/PR	47 (40–51.9)		0.38 (0.18–0.8)	
Side effects				
Yes	40 (27.6–51.3)	0.031	1 (reference)	0.09
No	20.3 (10.3–27.8)		1.83 (0.91–3.7)	

*CI* Confidence interval, *Cox* multivariate Cox regression analysis, *LR* log rank test, *mOS* median overall survival, *mPFS* median progression-free survival, *OR* odds ratio, *CR* complete remission, *PD* progressive disease, *PR* partial remission, *SD* stable disease

**Fig. 5:**
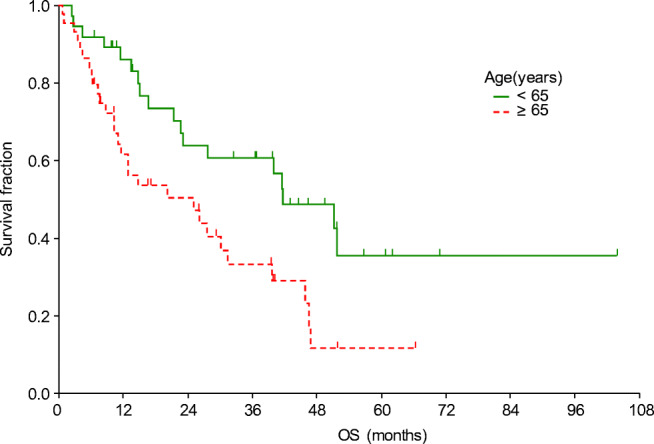
Overall survival curves according to patients’ age

**Fig. 6:**
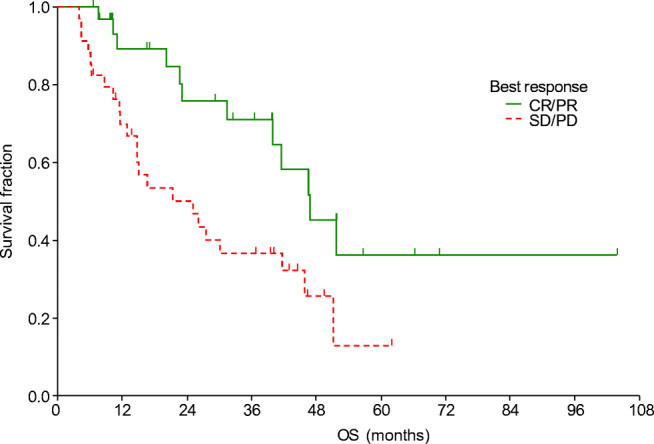
Overall survival curves according to the treatment response. CR, complete remission; PD, progressive disease; PR, partial remission; SD, stable disease

The OS was significantly influenced by age, presence of liver metastasis, experienced side effects, and best treatment response (Table 4, Figs. 5 and 6). Patients who were in good, moderate and poor prognostic groups had different median OS: 31.5 (23.2–46) (*N* = 35), 26.2 (12.9–40) (37) and 6.2 (2.9–6.2) (*N* = 7) months, respectively (*p* = 0.02).

## Discussion

The present study has been conducted at one site between 2013 and 2019 and includes the retrospective data analysis of patients with advanced renal cell carcinoma receiving PZ therapy.

Our patients with renal cancer were from the older age group (median age: 65.3 years), which is typical in RCC. The 1.9:1 male: female ratio also seems to be consistent with the international standards of male dominance in RCC [13]. Our patients presented 11.8 months median PFS and 30.2 months median OS, which is somewhat better than the results of registrational clinical trials (PFS: 9.2; OS: 22.9 months) [5, 6]. We have to mention that our patient cohort was more homogeneous, all of them underwent radical nephrectomy, and had pure clear cell histology. On the other hand, patient outcome observed in our study is similar to the results of published scientific papers (PFS: 4.6–12.4 months, OS: 21–29.9 months) [10, 14–16]. A Korean study presented a 40 months median OS [17], however, the authors did not explain the reasons for the unusually long survival.

With regard to the best response, the objective tumour response ratio of 33% which is similar to the registrational study (32.4%) [6], the COMPARZ study (31%) [10], or the PRINCIPAL study (30%) [15].

In Hungary, there are two types of reimbursed treatments available for advanced renal cell cancer in first line: sunitinib and pazopanib. The prospective, randomized non-inferiority COMPARZ study presented their efficacy to be close to identical [9, 10] Other retrospective studies showed similar [17] or contradictory results [16]. While according to the PISCES [11] and a Korean retrospective study [17], the PZ therapy is better tolerated by the patients. In the present study, the PZ treatment was well tolerated, severe (grade 3/4) side effects were rarely reported (only 14%), while 43% of patients did not present any adverse effect. The most common side effects were diarrhoea (35%), fatigue (17%), hypertension (14%), nausea/vomiting (10%), and decreased liver function (7%), while the most commonly reported side effects in the registrational trial were diarrhoea (52%), hypertension (40%), hair colour change (38%), and nausea (26%). Decreased liver function was present in 53% of patients [6]. A suggested potential reason for this controversy is that in the everyday practice, the side effects are not as systematically registered as they are in the randomized, prospective clinical trials. Twenty-five percent of patients had dose reduction, which refers to the frequency of the side effects. Higher or similar ratios have been published in the literature: in the comparative study of Motzer et al. (44%) [9], a Korean study (41%) [17], a Hungarian study (28.9%) [14], while in the PISCES study, the reported ratio was 13%, however in that study PZ was administered only for 10 weeks. The low dose reduction ratio also refers to good tolerability.

In this study the factors influencing survival were also investigated. Improved PFS and OS were observed in patients who did not have liver metastasis (*p* = 0.003 and *p* = 0.0004, respectively). The significance of liver metastasis was also highlighted by Kim et al. [17], nevertheless in their study, bone metastasis also caused worse outcome. Improved outcome was presented in our patients with complete or partial tumour response (*p* = 0.007). Similarly to other TKI therapies [18–20], the presence of side effects was a strong predictive marker of significantly longer PFS: all side effects (*p* = 0.0001), diarrhoea (*p* = 0.016). Dose reductions were observed to result in improved PFS (p = 0.016), which also indicates the significance of side effects. Increased OS was detected in patients who were under 65 years old (*p* = 0.008), presumably because co-morbidities and drug-drug interactions complicate the treatment of the elderly.

According to the literature, our study analysed the highest patient number from one centre, which is the strength of this study, meanwhile the weakness of the analysis is its retrospective nature.

Based on the present data, first-line pazopanib therapy for advanced RCC, even in unselected patients, is suggested to be an efficient, well tolerated treatment. Both OS and PFS were significantly improved by the absence of liver metastases. The presence of side effects proved to be an independent predictive marker of PFS, while younger age (<65 years) and objective response were independent markers of longer OS.
